# Passing Events and Topics

**Published:** 1922-03

**Authors:** 


### PASSING EVENTS AND TOPICS.

### THE TURN OF THE TIDE.

DESPITE the difficulties of the time, it is encouraging to note the individual instances of hospital prosperity. The first two to hand are drawn respectively from London and the provinces. Thus, the Royal Westminster Ophthalmic Hospital has increased its income by nearly £1,500, though legacies have been much reduced; it has also increased its expenditure only by £270, and ended the year less than £200 to the bad on current account, with a total overdraft of £840. If the tide continues in this direction, the hospital should be prosperous and self-supporting as a modest legacy even now would secure. At Norwich, too, we are glad to see that the income of the Norfolk and Norwich Hospital last year reached a record figure. For the first time in the past three years it more than covered the expenditure, and by a small sum the overdraft of £28,000 has been reduced. Expenses are expected to be lower, and as the contributors' scheme has realised £11,280, and can still be extended, it may be hoped that all legacies can be used, as they ought in theory, to reduce the debt. It will be noticed that these two examples can be challenged as exceptional. They only show the turn of the tide. But by the time that the peace shall have lasted as long as the war it seems reasonable to hope that the hospitals will have the worst of their difficulties behind them.

### ORGANISED CONTRIBUTIONS IN SCOTLAND.

AFTER a year's trial it is gratifying to recall the progress made in improving the organised contributions to the hospitals of Edinburgh and Glasgow. It is understood that from this source the Glasgow Royal Infirmary has received £30,000 or almost double the total raised in the previous year. In Edinburgh the League of Subscribers is producing comparable results, though in both cities income still lags behind expenditure. But the great thing is to start such schemes. We believe that every endeavour of the kind shows increasingly better results, and that, unlike other forms of subscription, weekly collections do not relapse or even remain stationary. The best results come from a scale of a penny in the pound, though at Glasgow the proportionate rise is less on wages above 60s. a week, and sixpence is the limit. In Glasgow the districts follow the Parliamentary divisions, but a good deal of steady work must yet be done before all the public offices even, let alone all the shops and offices, become contributors.

### THE LONDON HOSPITAL.

AT a time of closing and rumours of closing hospital wards, it is cheering to learn that the London Hospital is about to reopen the 200 beds shut down last May. An American visitor, in gratitude for the services rendered to his wife, made a gift of £5,000, with the promise of a further £5,000 if £10,000 were forthcoming before the end of 1921; the whole to be devoted to the establishment of a department for the treatment of serious complications arising out of gynæcological and maternity cases. At the last moment the condition was fulfilled, the Ministry of Health making a grant of the necessary £10,000. Mr. E. W. Morris, the House Governor of the London Hospital, states that as the financial position at the end of the year was better than it had been expected to be, the 200 beds would have been reopened in any case. The fact that this generous gift is to be devoted to a special purpose does not affect its value to the hospital and its patients, for the department has long been needed, and its establishment would have made a heavy call on the general funds.

### ROYAL WEDDING GIFTS.

FUNDS with a personal and a local object are being started all over the country to show the public approbation of Princess Mary's wedding. They have, here and there, been criticised, on the ground that it is a poor compliment to a friend to give a present to someone else upon the friend's marriage. But this will not stand a moment's examination. Princess Mary cannot be the personal friend of half the nation, and the starting of hospital funds in honour of her marriage is a recognised way of improving the occasion, and giving public expression to a public sentiment which from the nature of the case cannot express itself in a wholly personal way. If no more of these funds are started than local impulse warrants, they are to be commended, and if a grand total is reached for the hospitals, who will be more delighted than the Princess at having supplied the incentive?

### ALMONERS OF THE POOR-BOX.

MR. CECIL CHAPMAN, the well-known magistrate of Clerkenwell Police Court, threw some welcome light upon the problem of wisely administering the poor-box and other relief in a recent letter to the "Spectator." He described the work of a Police Magistrate in this regard as that of an almoner, and said that if certain C.O.S. rules were followed "it is possible to make a small fund of some £250 to £300 a year do the work of double that amount when carelessly administered." We can well believe it, and sympathise with Mr. Chapman's plea that the society which evolved these rules should be better supported, so that more of its district local committees might be formed. "If every city and borough," he concludes, "had its social service association, supported by private endeavour and voluntary contributions, the necessity for public assistance would gradually disappear, and not only would rates and taxes be diminished, but all the demoralising influences of public doles would be avoided." There is much good sense in this; but it is right to remember that the C.O.S. would naturally commend itself to a magistrate, since its rigid rules and tendency to regimentation are akin, in spirit, to the legal enactments which the magistrate has to administer. For this reason we do not share Mr. Chapman's regret that the C.O.S. has been often harshly criticised. Its activities, like those of the police, which they sometimes resemble, are valuable in proportion to the healthy criticism which they receive. A now famous man has left on record his experience in early days with the Society. That shows as nothing else could that if charity needs organisation, organisation is the better for charity.

### MEDICAL EDUCATION IN WALES.

IN accordance with the pound-for-pound principle applied in aiding the voluntary hospitals, the grant of £5,000 per annum offered by the Government to the Welsh National School of Medicine is conditional. A capital sum of £100,000, or its equivalent in annual income, must be raised by public subscription before August, 1922, or the Government offer will lapse. An appeal for subscriptions, signed by Lord Kenyon and Dr. A. H. Trow, Pro-Chancellor and Vice-Chancellor respectively of the University of Wales, of which the Medical School is part, is supported by a letter from the Prime Minister. Mr. Lloyd George points out that hitherto Welsh students who have done their preliminary training at the University College of South Wales and Monmouthshire have had to pursue their further training for the final degrees at University College, London, Edinburgh, Liverpool, or elsewhere, and he appeals to Wales to endow and equip a national school of her own. Mr. Gwilym Hughes, of Cardiff, organising secretary of the campaign to raise the £100,000, was the first chartered secretary of the King Edward VII. Welsh National Memorial Association for the Prevention, Treatment and Abolition of Tuberculosis. Donations to the Appeal Fund should be sent to him at the Welsh National School of Medicine, Cardiff. It will be remembered that the Welsh National School of Medicine was established last October. The King Edward VII. Hospital at Cardiff has placed all its beds at the disposal of the school for tuition purposes. A Physiological Block and Department of Public Health have been or are being built, and Chairs of Preventive Medicine and of Tuberculosis have been founded.

### THE NORWICH PROVIDENT SCHEME.

A NEW scheme has been started to help the finances of the Norfolk and Norwich Hospital. It is designed to gain the support of the smaller tradesmen and farmers who lie outside the scope of the Saturday and Sunday Funds. Its method is that each such person shall contribute an annual sum of £1 1s., 15s., 10s., or 5s., and, in return, have the right to free treatment should occasion arise. A married man who subscribes a guinea can ear-mark half for the benefit of his wife, a decision which brought in many supporters. It does not appear that the dependants of subscribers, unless a separate subscription is made in their name, are eligible. The plan belongs to the provident class which was devised successfully at Oxford and Brighton, and it has only to survive the test of London to be found applicable everywhere.

### THE SHEFFIELD SCHEME.

THE penny in the pound contributory scheme which Mr. Lamb is working hard to organise successfully in Sheffield, has met with certain criticisms. A flat rate is preferred in some industries, and the objectors ask what will be the position of those who have subscribed in the past, but do not join the present scheme which provides free treatment only to subscribers and their dependents. In the old days subscribers did not claim a right to treatment in return for their subscriptions, but gave because they believed in the voluntary system, and wished voluntarily to help the hospitals to help others. Consequently to advance a claim now in the light of past generosity is disingenuous; and even were this not the case, institutions can revise their rules and treat cases accordingly. So far as we know every hospital treats freely all urgent cases unable to contribute toward their maintenance.

### THE BIRTHDAY LEAGUE.

MR. R. J. COLES, Superintendent of King's College Hospital, who brought to London from Cambridge many ideas for raising hospital funds, is presumably the sponsor of the Birthday League lately launched so merrily by the medical students in the neighbourhood. At every house visited, for we hear of no exceptions, the names and birthdays of the residents were noted. They promised to send not less than 4s. to the hospital on each anniversary. This occasion brings them a birthday card from the hospital, with the following greeting: "This is to wish you Many Happy Returns of the day, and to remind you of your kind promise to send a birthday gift of 4s. and upwards as a thank-offering." Members of the League live as far away as New Zealand, and the idea is working well. If the lettering were done in script, and a block made, the card would have a distinction of its own at a small cost. Writers of script are shy birds, but they can be found with a little inquiry.

### ANOTHER CARD TO STAMP.

HOSPITAL stamps, on a large scale at least, have not hitherto been popular in this country, though they have had a regular vogue abroad in connection with a variety of institutions. But a new use has been found for them in Birmingham, where the Children's Hospital has started a scheme of penny a week contributions from householders. Each is supplied with a card, containing fifty-two divisions. The hospital issues a special stamp which can be purchased from the collectors weekly. These collectors work under the committee in charge of each district; and the stamps purchased by 300

householders produce £60 a year, a sum sufficient to meet the difference between the present income of each cot and its cost. It will be interesting to see if the scheme succeeds. At all events the average householder has been much educated of late years in stamping Government cards of one kind or another.

---

### A HOME NURSING BENEFIT PLAN.

MEMBERS of Approved Societies seem to be anxious to secure for themselves better provision in sickness than hitherto, and to subscribe for these additional advantages. The so-called hospital benefit is already making headway, and now comes a scheme for securing home nursing. Approved by the Leicestershire Insurance Committee, the plan invites Approved Societies to approach their members that upon the payment of a capitation fee, plus 1s. 4d. per visit up to a total of 3s., and 5s. a week for subsequent attendance, they may secure the advantages of a nurse. The work is to be done by arrangement with the Leicestershire District Nursing Association and the Approved Societies. If adopted the scheme will start on April 1. It will be interesting to hear the opinion of district nursing associations upon it, and how far they consider it an improvement on the facilities already offered by themselves.

---

### THE LATE SIR ARTHUR LUCAS.

A VERY good friend to the Great Ormond Street Hospital for Sick Children has been lost to it by Sir Arthur Lucas' death. For thirty out of his forty years service on the Committee, he was its chairman, and it is only a year since he retired. His long life (he died at the age of 76) was full of interests. Travel, art, music, and engineering, with active hospital work, filled his days. It was on engineering and construction that he was particularly valuable to his hospital. He resembled the late Sir Perceval Nairne, chairman of the Seamen's Hospital, in two respects. Both were men unostentatious in the extreme; few but those with inside knowledge knew how much they accomplished for the voluntary system. Both men also were keen yachtsmen. Sir Arthur Lucas indeed is understood to have qualified as a master mariner. His thoroughness could hardly be better illustrated.

---

### WREXHAM'S WAR MEMORIAL.

AN important scheme to provide a more adequate hospital for Wrexham and East Denbighshire is hanging in the balance. So long ago as 1917 a new hospital in Wrexham was proposed, to take the form of a war memorial. Beside this proposal, the trustees of the late Mr. John Jones were empowered to build a hospital of 25 beds on the property of the Roseneath site, for which plan £50,000 and a site were held in trust. The trustees, the Wrexham Infirmary and the War Memorial Committee, attempted to join forces, and, the provisional consent of the Charity Commissioners having been obtained, the public was asked to provide another £50,000, that the joint scheme might be carried out. A time limit to raise the money was imposed, and has since been extended till September 30, 1922, but some £20,000 is still required, if the whole £100,000 is to be spent upon a new, central hospital. But the larger scheme is proving beyond the purse of its supporters, who are now involved in the thorny questions of adaptation and half-measures. This is disappointing, because the usual difficulty in these cases is to secure agreement between the different bodies, not to raise the money once their forces have been joined.

---

### A NURSES' HOME FOR BOSCOMBE.

MR. CHILD CLARK'S splendid gift of a Nurses' Home to Boscombe branch of the Royal Victoria and West Hants Hospital, Bournemouth, where the unlucky staff has been hitherto dispersed under six roofs, is only one expression of his work for hospitals. He was formerly connected with the Royal Southern Hospital, Liverpool, and when he came to Bournemouth twenty years ago he began to urge the amalgamation of its two hospitals which was eventually accomplished by the late Dr. Roberts Thomson. The architect, Mr. H. E. Hawker, has provided a lavatory basin with hot and cold water for each of the thirty-five bedrooms, and the new home must be one of the first to be so furnished. It is interesting to hear from the chairman, Dr. A. Heygate Vernon, that before long the working classes will be providing half the cost of the hospital's maintenance.

---

### A COMING AMALGAMATION.

THE committees of the Sunderland Royal Infirmary and the Monk Wearmouth and Southwick Hospital have agreed to the principle of amalgamation. The plan, often proposed, is now being discussed in detail, but it is over the details that difficulties generally occur. One of the proposals is, after amalgamation, to build a new hospital on the Ashville Estate, Monk Wearmouth; but, on the face of it, such a plan would repeat the number of institutions and staffs which amalgamation might be expected to reduce.

---

### INFLUENZA CLOSES HOSPITAL.

HOSPITAL staffs are not immune from influenza, but few have suffered so severely as the recent victims at the Royal Sussex County Hospital, Brighton, which had to be closed temporarily because sixty-two members of the staff and patients fell ill. Since Christmas the staff has lost for varying periods the services of two resident medical officers, five sisters, twenty-nine nurses, seven maids and two porters, a total of forty-five. Seventeen patients were also ill. The hospital, therefore, had to be closed not only to visitors, but to patients, a serious but necessary step which perhaps has made the public keenly aware of the benefits ordinarily to be had for the asking. This is something like a record for hospitals, even in the post-war epidemics.

